# Supplementary material for: Heat guiding and focusing using ballistic phonon transport in phononic nanostructures
Source: Nat Commun. 2017 May 18;8:15505. doi: 10.1038/ncomms15505 (PMC5454390; doi:10.1038/ncomms15505)
Supplement: Supplementary Information — Supplementary Figures, Supplementary Notes and Supplementary References [file ncomms15505-s1.pdf]

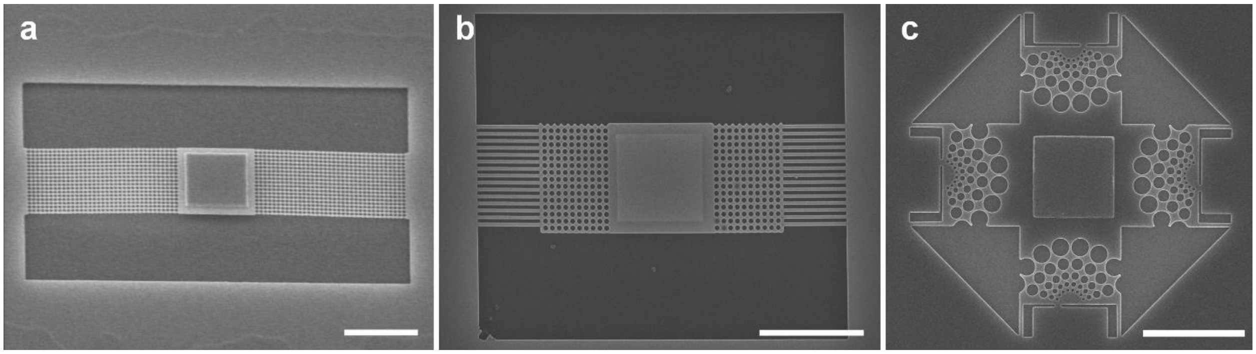

Supplementary Figure 1 | SEM images of typical samples. (**a**) Tilted view of an aligned sample, (**b**) top view of a nanowire-coupled sample and (**c**) top view of a converging lens sample. Scale bars are 5 µm.

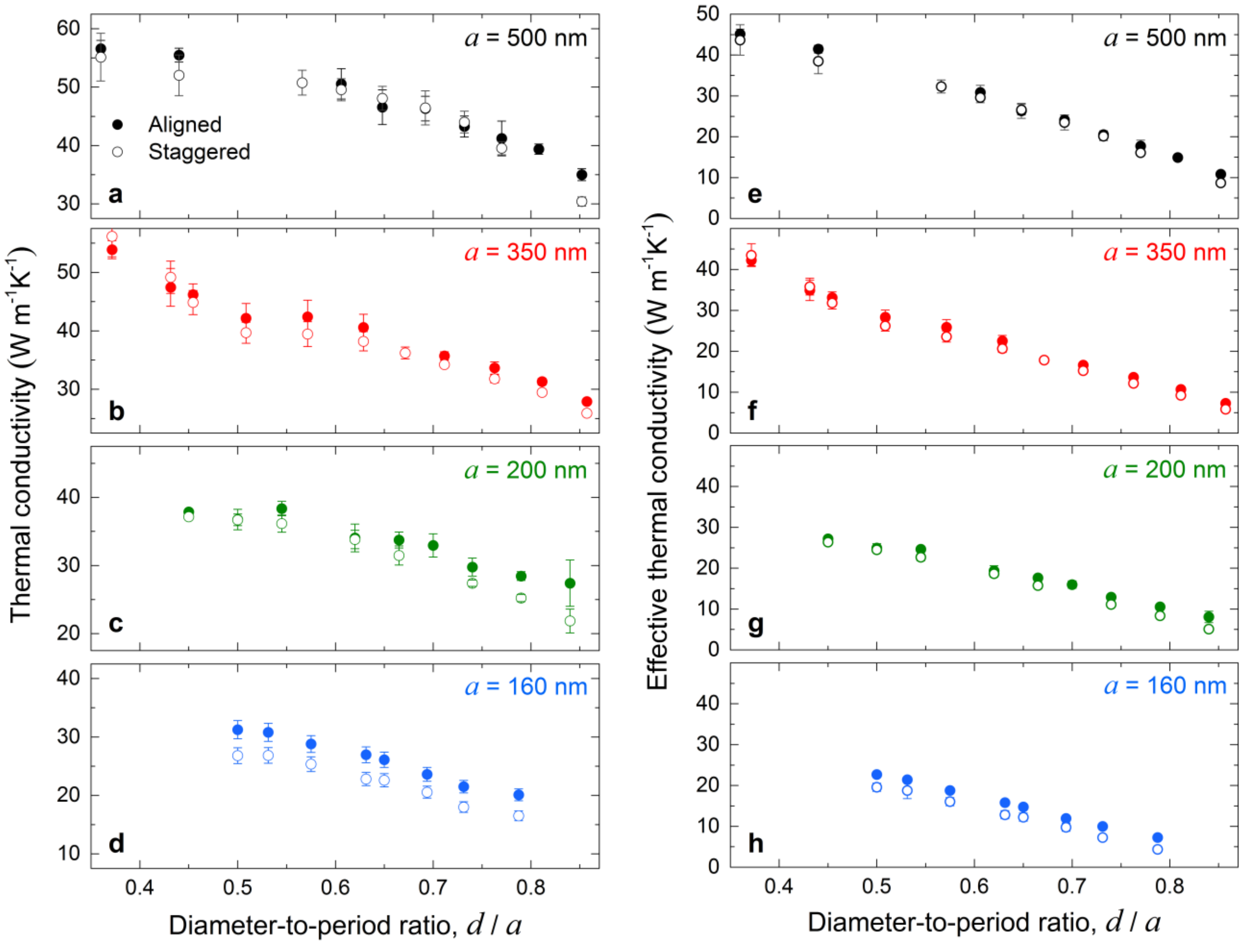

Supplementary Figure 2 | Thermal conductivity of the phononic crystals. Thermal conductivity and effective thermal conductivity measured at 300 K on aligned and staggered phononic crystals with periods of 500, 350, 200 and 160 nm, as a function of diameter-to-period ratio. Error bars show s. d. in different measurements.

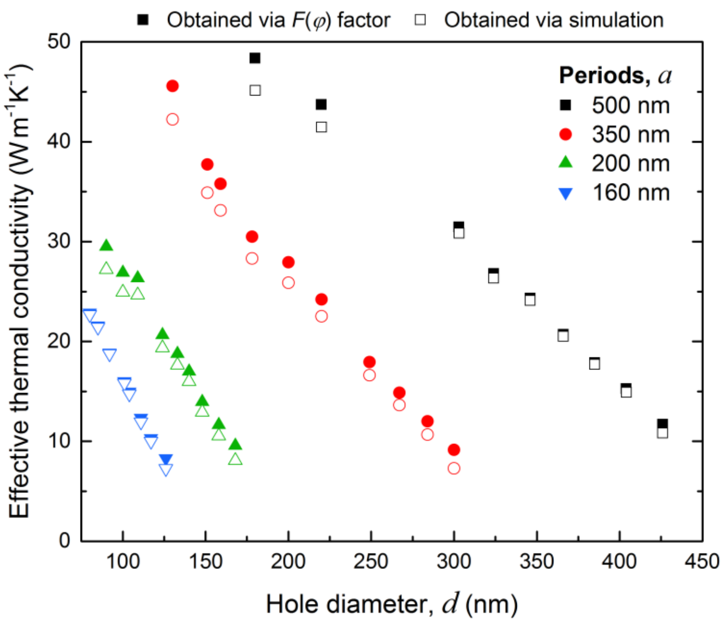

Supplementary Figure 3 | Effective thermal conductivity. Comparison of effective thermal conductivity (κ_{eff}) obtained via FEM simulations and via correction factor $F(\varphi)$ of aligned phononic crystals with different periods.

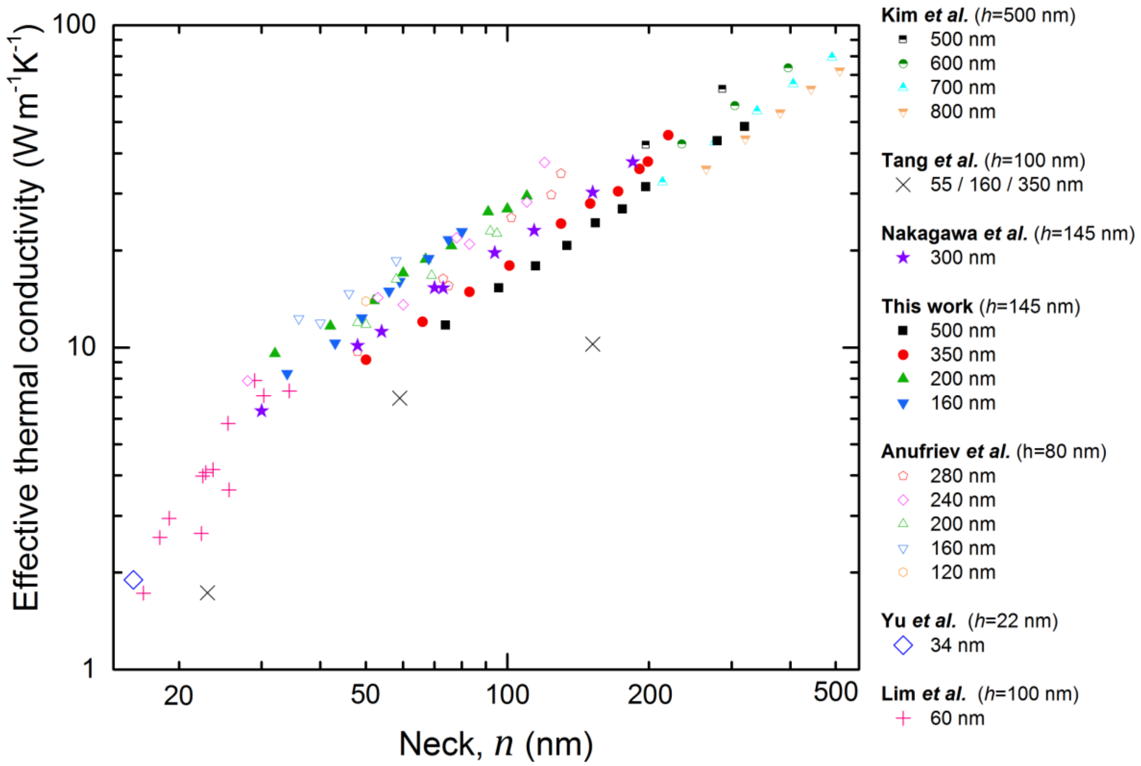

Supplementary Figure 4 | Effective thermal conductivity in the literature. Comparison of effective thermal conductivity (κ_{eff}) obtained in this work and in the literature on phononic crystals of different periods and thicknesses (h) plotted as a function of neck (n).

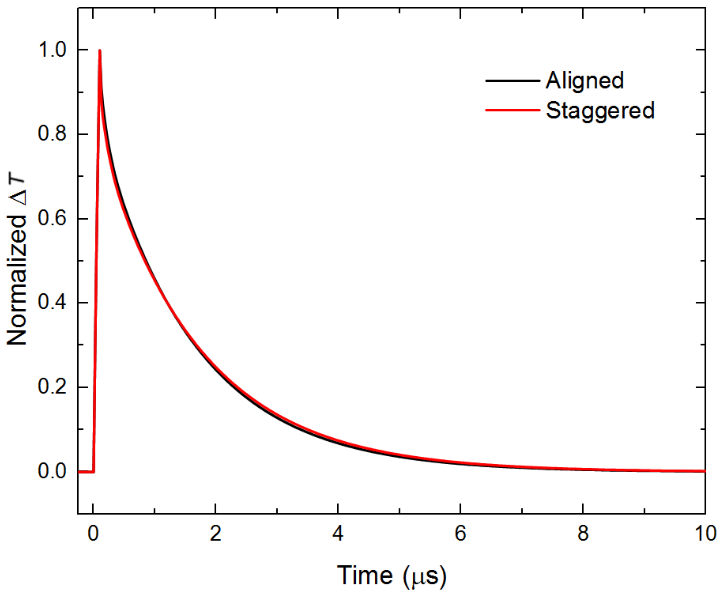

Supplementary Figure 5 | Simulated decay curves. The curves for aligned and staggered structures (a = 160 nm and d = 126 nm) show a good match, which proves that the alignment of holes is unimportant in the Fourier law approximation. The largest difference, obtained for the largest hole diameter in the smallest period, remains below 5%. Thus, the experimentally observed difference between the decay times of aligned and staggered lattices comes from a non-diffusive behavior. In the case of nanowire coupled and uncoupled samples, we also found no difference in decay times in the Fourier law approximation (not shown).

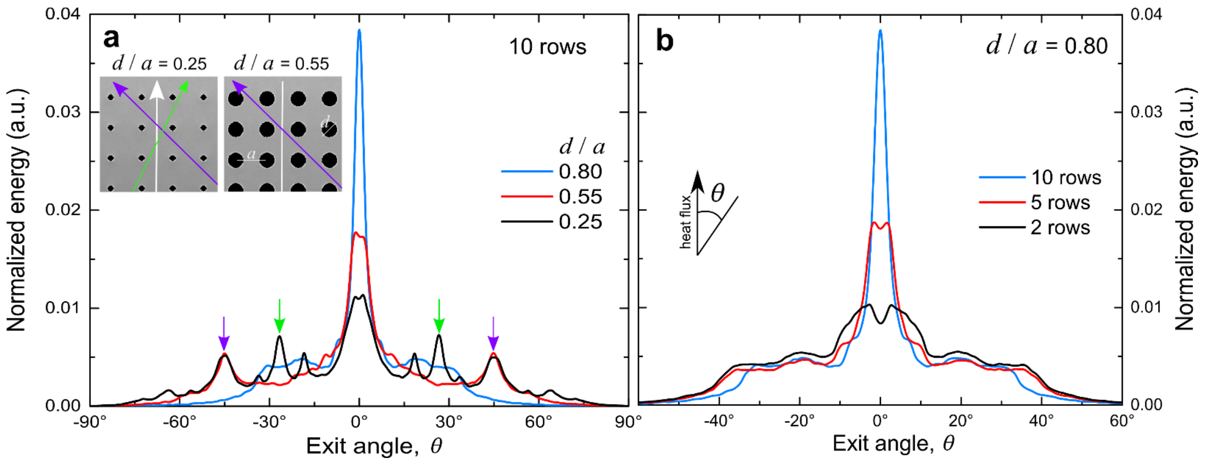

Supplementary Figure 6 | Heat flux directionality. Evolution of heat flux directionality in aligned lattices as a function of the diameter-to-period ratio d / a and the number of rows of holes. (**a**) As d / a ratio is increased, the different direct passageways through the phononic crystals (depicted by the peaks) becoming blocked until only the central vertical path remains. (**b**) The higher the number of rows, the sharper the central peaks become for a given d / a ratio.

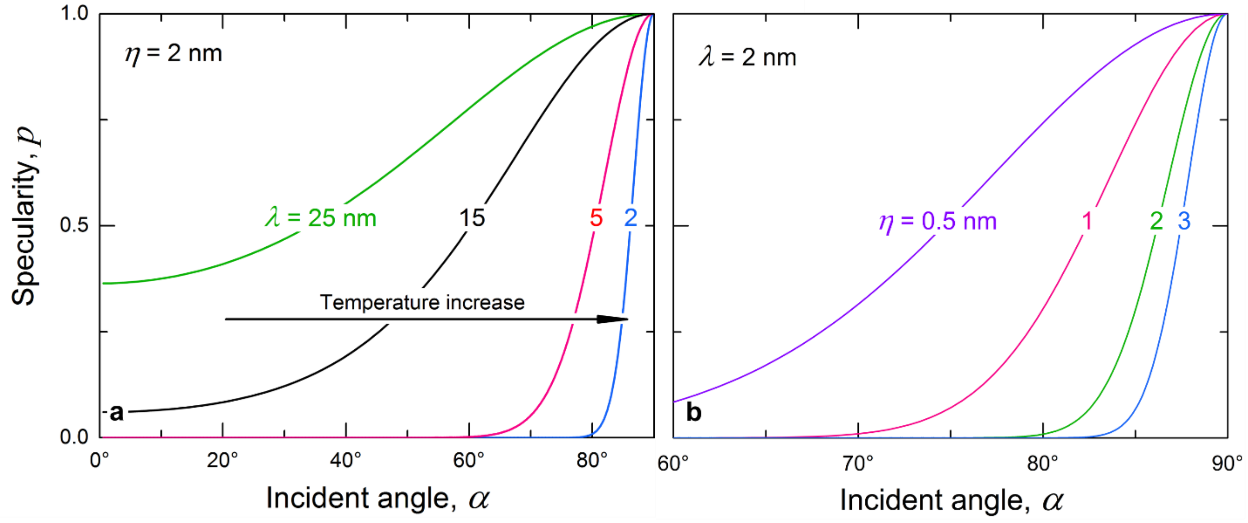

Supplementary Figure 7 | Specularity *vs.* wavelength and roughness. Specularity parameter increases with the incidence angle, phonon wavelength and rms surface roughness.

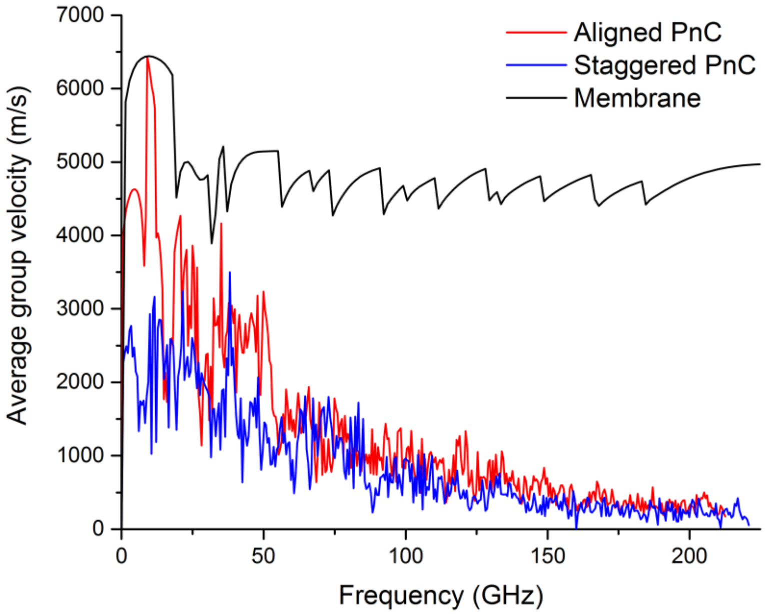

Supplementary Figure 8 | Phononic effect in different lattices. Reduction in the group velocity spectra, calculated in purely coherent scattering approximation[1], is very similar in aligned and staggered lattices. Thus, we conclude that even if some coherent effects are present at low temperatures, they affect aligned and staggered lattices alike.

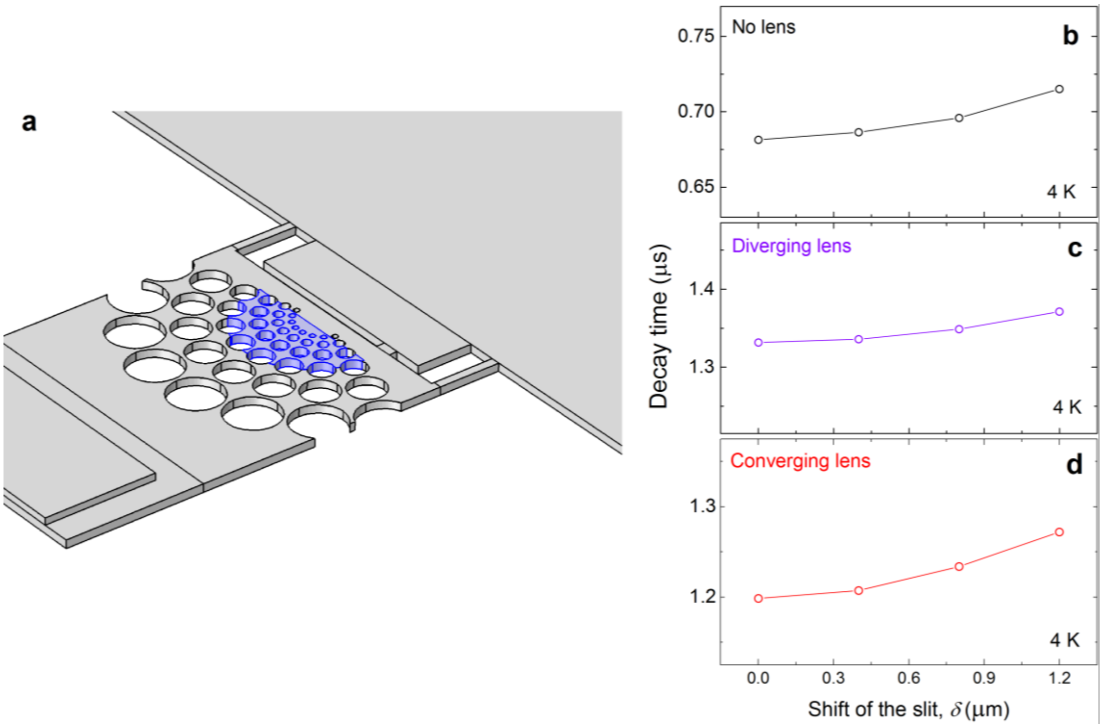

Supplementary Figure 9 | FEM simulations of thermal lenses. (**a**) Simulated structure with the region of lower thermal conductivity. To account for the different regions of thermal conductivity, we set 3.5 times lower thermal conductivity in the blue region, according to our previous work[2], and twice lower thermal conductivity in the wires and the slit. (**b-d**) Obtained trends of decay times seem to be very similar for converging lens, diverging lens, and even reference membranes. This resembles neither our experimental results nor Monte Carlo simulations, shown in the main text, as the reference samples experimentally show flatter trends whereas converging lens has a steeper trend. Moreover, whereas FEM trends are nearly the same for all three types of samples, the experimental trends are clearly different. Thus, we conclude that the experimental results cannot be explained in terms of purely diffusive transport.

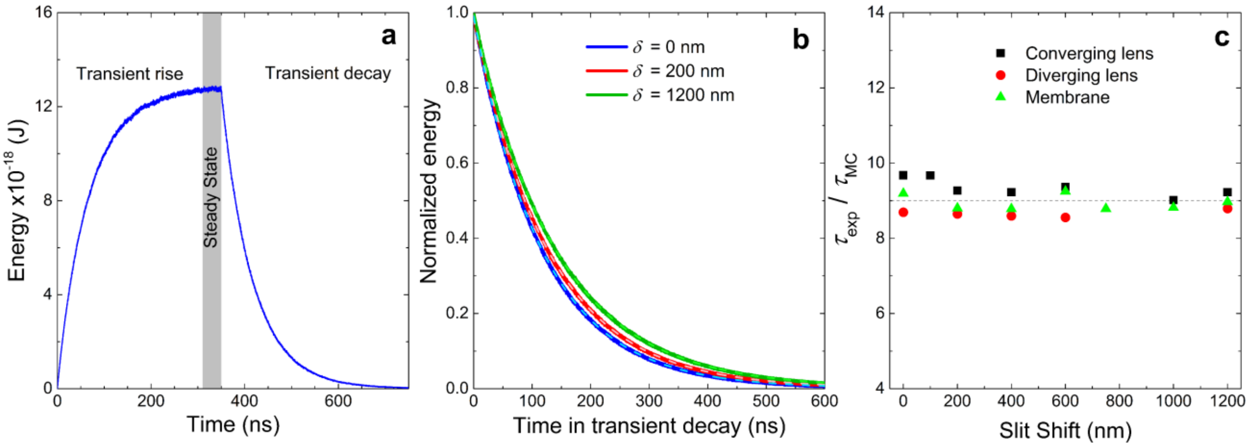

Supplementary Figure 10 | Simulated decay times. Typical evolution of energy of the heated part of the silicon as a function of time. (**a**) At the beginning, adding phonons increases the energy (transient rise). Then, the steady state covers ~40 ns (40,000 time steps). When no more phonons are added, the energy decreases in a transient decay. (**b**) The energy in the transient decay part is normalized and fitted with an exponential decay, displayed in dashed lines, to obtain the decay times. (**c**) The ratio of the experimental decay times over the simulated decay times is a constant. Thus, the MC code reproduces correctly the experimental tendencies.

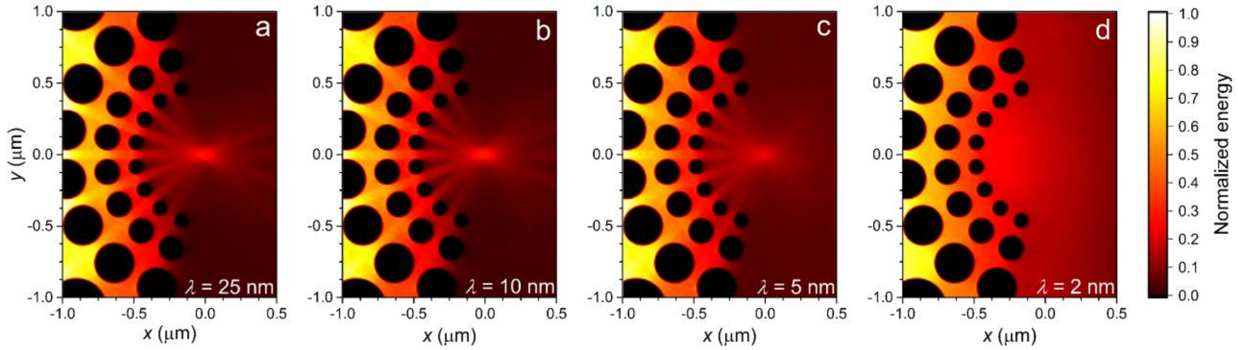

Supplementary Figure 11 | Heat focusing *vs.* phonon wavelength. We carried out simulations with monochromatic emission of phonons following the dominant phonon wavelength as $\lambda_{\mathrm{d}} = hv/(2.82k_{\mathrm{B}}T)$. Thus, all phonons were emitted with a single wavelength. Panels **a**, **b**, **c** and **d** show the evolution of the hot spot for phonons with wavelengths of 25, 10, 5 and 2 nm, respectively. As the wavelength is reduced, the hotspot blurs and gradually disappears. According to the dominant wavelength formula above with $v = 6000$ m s^{-1}, we obtain $T(\lambda_{\mathrm{d}} = 25\ \mathrm{nm}) \approx 4$ K, $T(\lambda_{\mathrm{d}} = 10\ \mathrm{nm}) \approx 10$ K, $T(\lambda_{\mathrm{d}} = 5\ \mathrm{nm}) \approx 20$ K and $T(\lambda_{\mathrm{d}} = 10\ \mathrm{nm}) \approx 50$ K. However, this formula is valid only at low temperatures because it is based on the Debye approximation.[3] Indeed, even at room temperature the phonon wavelengths are estimated to be still in the 0.5 – 5 nm range[4]. Since we can still observe the hotspot for λ_{d} = 5 nm, it may explain the experimentally observed dependence on the slit position even at 300 K in the inset of Fig. 5g in the main text.

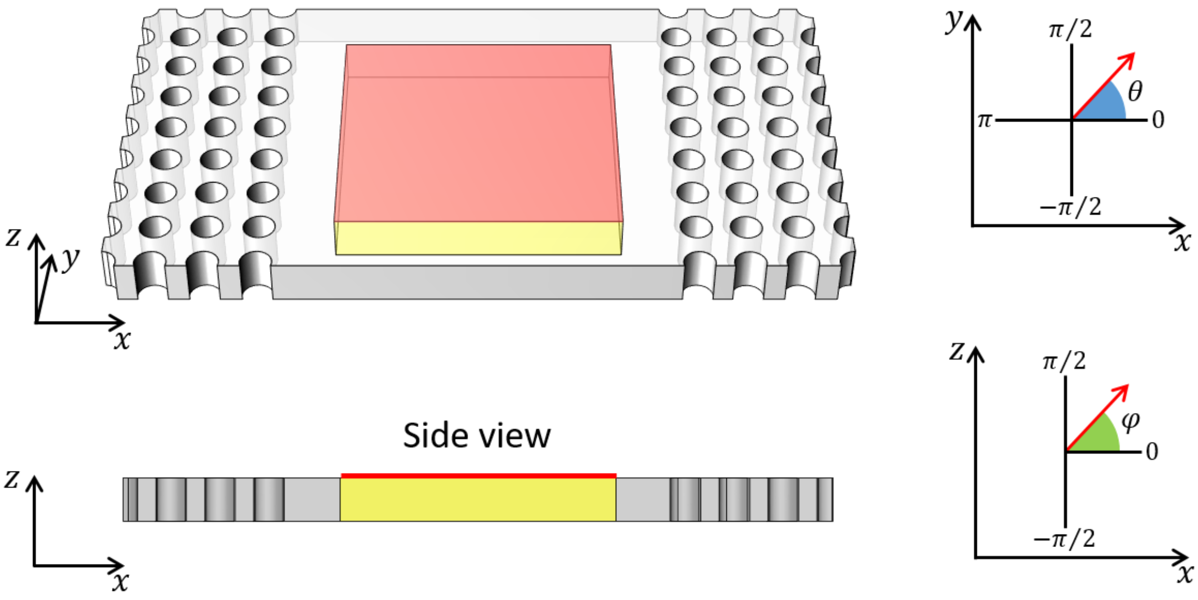

Supplementary Figure 12 | Schematic of Monte Carlo simulation model. Representation of the simulated nanostructures with adopted angles conventions. The red square depicts the starting area of the phonons. The yellow box is the volume where we count the energy that is equivalently probed experimentally by the metal pad.

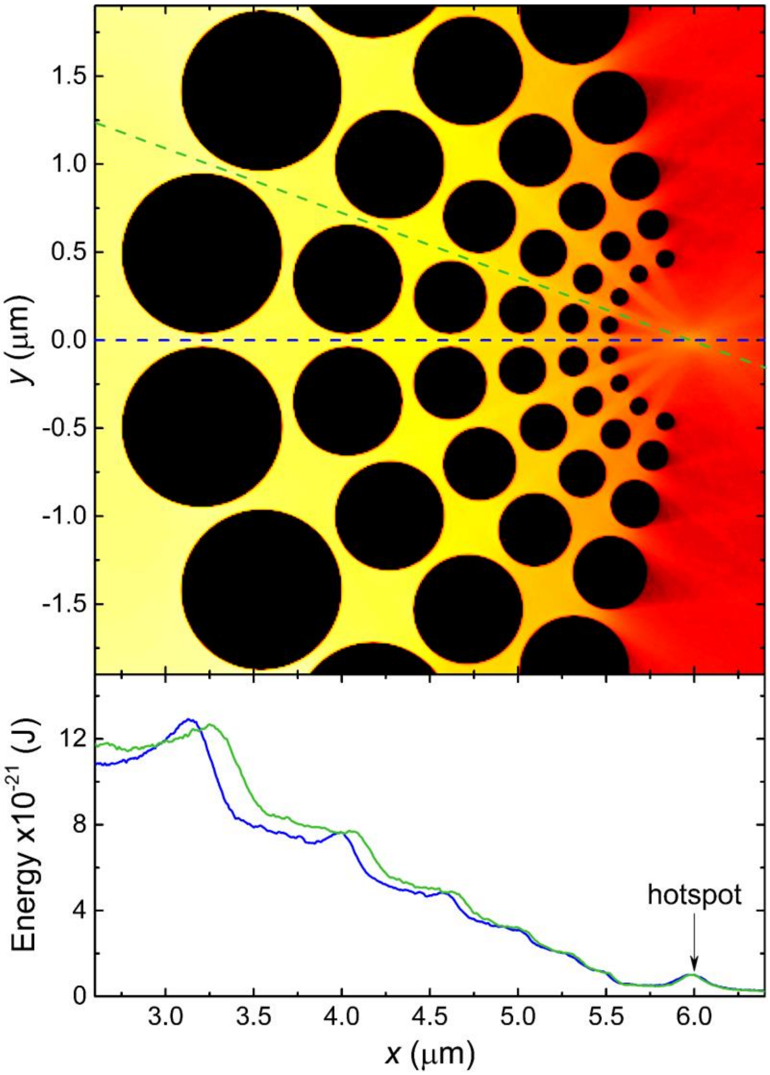

Supplementary Figure 13 | Full map of the energy in converging thermal lens together with energy profiles along two dashed lines. The intensity of the hotspot is approximately ten times lower than the heated region. The increase of energy observed for x = 3.2 μm and x = 4.0 μm correspond to the regions just before the passage between two holes where many phonons are backscattered on the boundaries of the holes. This effect weakens as a function of the number of holes the phonons cross because the phonons gradually acquire the directionality of the direct passage between the holes.

Supplementary Note 1. Thermal conductivity in aligned and staggered samples

To extract the thermal conductivity of our samples from the measured values of decay time, we performed FEM simulations using Comsol Multiphysics. The measured decay times are strongly dependent on the geometry of the structure and placement of the holes; thus, to extract the thermal conductivity, we created FEM models with exact replica of each investigated structure (including holes) and simulated the same experiment as that conducted with the μ-TDTR setup. The excitation is simulated by a heat flux pulse with Gaussian distribution on the aluminum pad. Using the thermal conductivity of the phononic crystal region as a free sweeping parameter, we simulate heat dissipation through the structure and monitor changes of temperature in the metal pad. The values of thermal conductivity[2,5] of Al pad and 145-nm-thick Si membrane are 237 and 75 W $m^{-1}K^{-1}$; the values of the heat capacity of Al and Si are 904 and 700 J $kg^{-1}K^{-1}$. Once the simulated decay curve matches the one measured experimentally, we record the value of thermal conductivity[6].

Supplementary Figure 2a-d show values of thermal conductivity obtained for all samples. The difference between aligned and staggered structures grows as the period is decreased. The largest difference is observed in the structures of 160 nm in period and high diameter-to-period ratio; the corresponding thermal conductivities are 20.1 and 16.5 W m^{-1} K^{-1} for the aligned and staggered lattices, respectively.

Note that these values of thermal conductivity (κ) represent thermal conductivity of the material between the holes, in contrast to the effective thermal conductivity of holey material (κ_{eff}), which is often used in the literature. To compare our data to the literature, we also extracted the effective thermal conductivity by performing the same FEM analysis as above, but using models without holes in simulated membranes. The obtained values of effective thermal conductivity are reported in Supplementary Figure 2e-h. We find that these two types of thermal conductivity correspond approximately as $\kappa_{\text{eff}} = F(\varphi)\cdot\kappa$, where $F(\varphi)$ is the correction factor that takes into account the volume reduction. In porous structures, this factor can be expressed[7,8] as $F(\varphi) = (1 - \varphi) / (1 + \varphi / 2)$, where φ is the porosity. Supplementary Figure 3 shows a good

agreement between the values of effective thermal conductivity, that were obtained as $\kappa_{\mathrm{eff}} = F(\varphi)\cdot\kappa$, and those obtained by the FEM simulations. Note that the factor in the form of $F(\varphi) = (1 - \varphi) / (1 + \varphi)$ can also be found in the literature and also gives a satisfactory agreement (not shown).

Next, we compare these data to experimental values of effective thermal conductivity in phononic crystals found in literature: Kim et al.[9], Anufriev *et al.*[2], Nakagawa *et al.*[10], Tang *et al.*[8], Yu *et al.*[11] and Lim *et al.*[12]. For clarity, where several lattices are studied, we plot only the data on square lattice. Supplementary Figure 4 shows all the data plotted as a function of neck – the distance between two neighboring holes.

Although the plot is logarithmic, most of the data points seem to form a common trend despite the fact that phononic crystals studied in the literature have a very different thickness (h). The data obtained in this work are in good agreement with our previous measurements[2,10] on phononic crystals of 80 and 145 nm in thickness. Although Kim *et al.*[9] measured phononic crystals with generally larger dimensions, their data points seem to continue the same trend formed by our data. Yu *et al.*[11] and Lim *et al.*[12] studied extremely small phononic crystals fabricated using block copolymer lithography and their structures displayed very small thermal conductivities, yet they too seem to follow the same trend. The data from Tang *et al.*[8], however, is lower than all other data, probably due to the very high surface roughness of their samples.

Supplementary Note 2. Impact of diameter-to-period ratio and row number on heat directionality

In the main text we have shown that phonons acquire a well-defined directionality along the heat flux (θ = 0°) when they exit the phononic crystal with ten rows of aligned lattice with the diameter-to-period ratio d / a = 0.85 (Fig. 3c). As phonons can propagate only in between the holes, both the diameter-to-period ratio and the number of rows impact the heat flow directionality. Here, we carry out Monte Carlo simulations to observe these dependencies.

Supplementary Figure 6a shows that for a low d / a ratio, set here as d / a = 0.25, several direct passageways are available for the phonons through the phononic crystal (shown by arrows in inset). Because of the ballisticity, phonons can propagate more easily in these directions, creating corresponding energy peaks. As d / a ratio increases, some of the direct passageways are blocked. For example, as d / a was increased from 0.25 to 0.55, the peak around 27°, indicated by a green arrow, disappeared. Finally, only one direct way with θ = 0° remains for high enough d / a ratios.

The number of rows also contributes to the directionality (Supplementary Figure 6b). At least five rows are required for the central peak of the exit angle distribution to dominate over the other directions. The dip observed at θ = 0° when just a few rows are present (also visible in Supplementary Figure 6a for the low d / a ratios) appears because phonons have slightly lower probability to travel strictly parallel to the passage between the holes than to travel at some angle due to the scattering on holes. As the number of rows is increased, phonons are concentrated along θ = 0°, hence repopulating this region in the steady state regime where we take our data. A single sharp peak therefore requires simultaneously a large d / a ratio (> 0.75) to block the diagonal direct passages and at least several rows of holes to retain the single sharp peak in the steady state.

Supplementary Note 3. Probability of specular surface scattering

In the main text, we estimate the probability of specular surface scattering (p) as

$$p = exp\,(-16\,\pi^2\,\eta^2\cos^2\alpha\,/\,\lambda^2) \qquad (1)$$

where λ is the phonon wavelength, η is the root mean square surface roughness (in our case η = 2 nm) and α is the normal incidence angle[13]. Supplementary Figure 7a shows how this specularity parameter increases with the incidence angle and wavelength. The wavelengths of 25 and 2 nm approximately correspond to the dominant phonon wavelengths at 4 and 300 K, respectively. Thus, not only at 4 K but even at room temperature some phonon can reflect specularly if they approach surfaces tangentially.

The specularity parameter also depends on the surface roughness. In this work, we only approximately estimated the surface roughness of η = 2 nm, however, this is a rather upper boundary of this parameter. Supplementary Figure 7b shows that the specularity strongly depends even on slight changes of surface roughness. Thus, if the roughness is overestimated and actual roughness is smaller by even just one nanometre, the probability of specular reflections greatly increases.

However, note that Supplementary equation 1 only estimates the probability of specular surface scattering and is valid only in the absence of surface correlations. The role of correlation was studied by Maurer *et al.*[14], Feser *et al.*[15], Ghossoub *et al.*[16] and Lim *et al.*[17]. Whereas the data from Lim *et al.*[17] seem to show an increase of the thermal conductivity when the correlation length increases, the data from Feser *et al.*[15] and the latest measurements by Maurer *et al.*[14] show no significant impact of the correlation length. However, the roughness induced by our fabrication process is most probably random and the correlation should be absent, thus using Supplementary equation 1 to estimate the specularity parameter seems reasonable.

Supplementary Note 4. Simulations of decay time

Monte Carlo algorithm traces phonons and records their energy in time. Thus, we are able to capture the different dynamics of the system and especially the decay of energy that is experimentally measured by our μ-TDTR system. Supplementary Figure 10a shows a typical evolution of the energy inside the silicon region under the metal pad. Initially, the structure is at thermal equilibrium at 4 K. At the time $t = 0$, the algorithm starts adding energy at the top of the silicon membrane, in the area corresponding to the metal pad, imitating the heating by the pump laser. The created phonons propagate in the structure until they reach the cold reservoir or until the end of the simulation. As phonon propagate, they are scattered by the geometrical boundaries and internal processes (impurities and normal phonon-phonon). At every time step, the algorithm adds new phonons according to the power that was set for the pump laser until the end of the pulse. The energy of the new phonons adds to the energy already present in the heated area at the given time.

At first, this procedure results in an increase of the energy (transient rise) in the heated area. Then, as the system reaches an equilibrium between the phonons escaping and entering the heated area, the energy in the heated area saturates and the system turns into a steady state. This steady state region, shown in Supplementary Figure 10a, contains 40,000 time steps. The energy maps in the main text (Figs. 3a, 3c, 5b, and 5d) are produced with the average energy of the last 10,000 time steps of the steady state as described in Methods. Once the heat pulse is over, no more energy is added, and the energy is rapidly decreased (transient decay).

Like in the experimental procedure, to extract the decay time, the simulated energy is normalized to get an energy equal to one at the beginning of the transient decay. Examples of decay curves are given in Supplementary Figure 10b for the case of the converging lens with different positions of the slit. All the decay curves can be fitted with an exponential function $exp(-t/\tau_{\mathrm{MC}})$, where τ_{MC} is the Monte Carlo decay time. However, τ_{MC} cannot be directly compared to the experimental decay time τ_{exp} as the decay

times obtained in the simulation are much shorter than experimental values. Nevertheless, the ratio $\tau_{\text{exp}}/\tau_{\text{MC}}$ remains approximately the same for all samples, as shown in Supplementary Figure 10c. Thus, we conclude that although our simulations miss some background phenomena that evenly affect all the structures, simulations well reproduce the changes in decay time caused by changes in geometry.

Supplementary Note 5. Trajectories of the phonons in Monte Carlo simulations

Supplementary Figure 12 shows schematics of a simulated nanostructure and the convention of directions and angles that we use. To initialize a phonon we set three parameters: position, direction, and energy. The initial position of a phonon is taken in the red square on the top surface at the center of the structure by picking uniform random numbers x and y such that $x \in [-2:2]$ µm, $y \in [-2:2]$ µm while $z = 0.145$ µm is constant. The initial direction of phonon propagation is determined by randomly drawing the couple of angles $(\theta; \varphi)$, so that the in-plane angle θ is isotropic in $[-\pi; \pi]$ and the out-of-plane angle φ follows the Lambert's cosine law[18] in $[-\pi/2\,; 0]$ at $t = 0$. The energy follows a phonon Planck distribution given by

$$B(\omega, T) = \frac{3\hbar}{2\pi^2 v_{\text{D}}^3} \frac{\omega^3}{\exp\left(\frac{\hbar\omega}{k_{\text{B}}T}\right) - 1}\,, \tag{2}$$

where $v_{\text{D}} = 6000$ m s^{-1} is the group velocity, identical for all the phonons in the Debye approximation of the linear relation of dispersion, which is valid only below 20 K. The phonons coming from the cold reservoir are subtracted from those emitted by the heater to simulate only the phonons participating to the heat transfer.[3] The resulting distribution is then randomly reproduced using a Von Neumann rejection method. All the random numbers in the code are generated by using the Mersenne Twister mt19937_64 generator seeded with the clock of the computer.[19]

Then, the phonon moves in a straight line following the equations:

$$\begin{cases} x(t + \Delta t) = x(t) + \cos(\varphi)\cos(\theta)\,\Delta t \\ y(t + \Delta t) = y(t) + \cos(\varphi)\sin(\theta)\,\Delta t \\ z(t + \Delta t) = z(t) + \sin(\varphi)\,\Delta t \end{cases}, \tag{3}$$

where Δt is the time step, usually set to 1 ps. If one or several boundary collisions are detected during the time step, the program calculate the position(s) of the collision(s) with the boundaries and determine the new trajectory of the phonon by taking into account the roughness of each boundary (see Methods) with the specularity parameter (p) given by Supplementary equation 1. For each collision, a uniform random number $r \in [0:1]$ is picked. If $r < p$, the reflection on the boundary is specular and the phonon conserves its angle to the local normal to the surface; otherwise, it is a diffuse reflection and a new direction is drawn randomly following the Lambert's cosine law.

At the end of the time step, the phonon can undergo an internal diffusion as described in the methods section of the main text.

As phonons travel through the structure, the boundary scattering processes progressively shape the phonon direction. This is clearly visible in the θ and φ angle distributions, which depict the number of phonons (normalized) in each direction. The θ angle distributions are displayed in the main text and are dependent on the geometry of the system. A Von Neumann rejection method was implemented in order to directly use this distribution to simulate the starting angle φ in the nanowires for the Figure 4 of the main text. The same procedure was used to simulate the initial θ angle, shown Figure 3d in the main text.

The yellow box in the Supplementary Figure 12 represents the control volume to determine the energy in the central region of the nanostructure. The energy in this yellow box is assimilated experimentally to the temperature of silicon that is directly measured by the probe laser on the metal pad.

Supplementary References

1. Anufriev, R. & Nomura, M. Reduction of thermal conductance by coherent phonon scattering in two-dimensional phononic crystals of different lattice types. *Phys. Rev. B* **93,** 45410 (2016).

2. Anufriev, R., Maire, J. & Nomura, M. Reduction of thermal conductivity by surface scattering of phonons in periodic silicon nanostructures. *Phys. Rev. B* **93,** 45411 (2016).

3. Ramiere, A., Volz, S. & Amrit, J. Heat flux induced blueshift of dominant phonon wavelength and its impact on thermal conductivity. *AIP Adv.* **7,** 15017 (2017).

4. Esfarjani, K., Chen, G. & Stokes, H. T. Heat transport in silicon from first-principles calculations. *Phys. Rev. B* **84,** 85204 (2011).

5. Touloukian, Y. S., Powell, R. W., Ho, C. Y. & Klemens, P. G. *Thermophysical properties of matter - The TPRC data series. Volume 2. Thermal conductivity - Nonmetallic solids*. (1971).

6. Maire, J. PhD: Thermal phonon transport in silicon nanostructures. (The University of Tokyo, 2015).

7. Machrafi, H. & Lebon, G. Size and porosity effects on thermal conductivity of nanoporous material with an extension to nanoporous particles embedded in a host matrix. *Phys. Lett. Sect. A Gen. At. Solid State Phys.* **379,** 968–973 (2015).

8. Tang, J. *et al.* Holey silicon as an efficient thermoelectric material. *Nano Lett.* **10,** 4279–83 (2010).

9. Kim, B. *et al.* Thermal conductivity manipulation in lithographically patterned single crystal silicon phononic crystal structures. in *International Ultrasonics Symposium Proceedings* 1308–1311 (2011).

10. Nakagawa, J., Kage, Y., Hori, T., Shiomi, J. & Nomura, M. Crystal structure dependent thermal conductivity in two-dimensional phononic crystal nanostructures. *Appl. Phys. Lett.* **107,** 23104 (2015).

11. Yu, J.-K., Mitrovic, S., Tham, D., Varghese, J. & Heath, J. R. Reduction of thermal conductivity in phononic nanomesh structures. *Nat. Nanotechnol.* **5,** 718–21 (2010).

12. Lim, J. *et al.* Simultaneous thermoelectric property measurement and incoherent phonon transport in holey silicon. *ACS Nano* **10,** 124–132 (2016).

13. Soffer, S. B. Statistical model for the size effect in electrical conduction. *J. Appl. Phys.* **38,** 1710 (1967).

14. Maurer, L. N., Aksamija, Z., Ramayya, E. B., Davoody, A. H. & Knezevic, I. Universal features of phonon transport in nanowires with correlated surface roughness. *Appl. Phys. Lett.* **106,** 6–11 (2015).

15. Feser, J. P. *et al.* Thermal conductivity of silicon nanowire arrays with controlled roughness. *J. Appl. Phys.* **112,** 114306 (2012).

16. Ghossoub, M. G. *et al.* Spectral phonon scattering from sub-10 nm surface roughness wavelengths in metal-assisted chemically etched Si nanowires. *Nano Lett.* **13,** 1564–1571 (2013).

17. Lim, J., Hippalgaonkar, K., Andrews, S., Majumdar, A. & Yang, P. Quantifying surface roughness effects on phonon transport in silicon nanowires. *Nano Lett.* **12,** 2475–82 (2012).

18. Klitsner, T. & Pohl, R. Phonon scattering at silicon crystal surfaces. *Phys. Rev. B. Condens. Matter* **36,** 6551–6565 (1987).

19. Matsumoto, M. & Nishimura, T. Mersenne twister: a 623-dimensionally equidistributed uniform pseudo-random number generator. *ACM Trans. Model. Comput. Simul.* **8,** 3–30 (1998).
